# Deep Ocean Minerals Minimize Eccentric Exercise-Induced Inflammatory Response of Rat Skeletal Muscle

**DOI:** 10.3389/fphys.2018.01351

**Published:** 2018-09-28

**Authors:** Suchada Saovieng, Jinfu Wu, Chih-Yang Huang, Chung-Lan Kao, Matthew F. Higgins, Rungchai Chuanchaiyakul, Chia-Hua Kuo

**Affiliations:** ^1^Laboratory of Exercise Biochemistry, University of Taipei, Taipei, Taiwan; ^2^Graduate Institute of Basic Medical Science, China Medical University, Taichung, Taiwan; ^3^Department of Healthcare Administration, Asia University, Taichung, Taiwan; ^4^Department of Life Sciences, University of Derby, Derby, United Kingdom; ^5^College of Sports Science and Technology, Mahidol University, Bangkok, Thailand

**Keywords:** skeletal muscle, macrophage, fructose, TNF-α, IL-10, glutathione

## Abstract

**Background:** We have previously shown an accelerated recovery from muscle fatigue in men challenged by prolonged exercise after oral deep ocean minerals (DOM) supplementation. Here, we hypothesized a decrease in eccentric exercise-induced muscle inflammation in rats regularly consuming DOM-containing drinks (hardness 600 mg/L and fructose 11%).

**Methods:** Forty-seven male Sprague Dawley rats were randomized into 4 groups: Control (C, *N* = 12), Fructose (F, *N* = 12), Fructose+Exercise (FE, *N* = 12), and Fructose+Exercise+DOM (FED, *N* = 11). Since fructose is a commonly used ingredient in beverages, 11% of fructose was added as a vehicle of the study. Soleus muscles of rats were analyzed 24 h after an acute bout of downhill running following 9 weeks of DOM supplementation.

**Results:** Leukocyte infiltration and TNF-α mRNA of muscle in the FE group were 5 times and 4 times greater the F group, respectively, (*P* < 0.05). Both markers in the FED group were significantly lower than those in the FE group (*P* < 0.05). IL-10 mRNA of muscle in the F group was >eight fold greater than the C group (*P* < 0.05). The reduced glutathione (GSH) of muscle in the F group was 34% lower than that in the C group (*P* < 0.05). However, GSH levels were similar for the C and FED groups.

**Conclusion:** Prolonged fructose supplementation modulates inflammatory balance of rat skeletal muscle. The results of the study suggest that DOM can minimize eccentric exercise-induced inflammatory cytokine responses in rat skeletal muscle.

## Introduction

An increasing amount of paleobiological evidence suggests that deep oceans are the sites where life on earth originated (Gingerich et al., 2001; Leslie, 2015; Keller et al., 2017). This concept implicates an innate deficiency in nutritive complexity for terrestrial descendants who survived after evolutionary sea-to-land migration. More than 80 minerals and trace elements existing in the ocean water have been reported (Farrington, 2000). Compared with surface water inland and ocean where light is permeable, the nutritive advantage of deep ocean water (depth > 200 m) is the preservation of biogenic components to be extracted by photosynthesis of local living organisms (Othmer and Roels, 1973). With similar profiles in major minerals (e.g., magnesium, potassium, calcium, sodium, chloride, and sulfate ions), deep ocean water has demonstrated a superior effect compared to surface ocean water on improving vascular functions in rabbits fed a high cholesterol diet (Miyamura et al., 2004).

We have previously found an accelerated recovery from muscle fatigue in men consuming DOM drink during recovery after a prolonged exercise (Hou et al., 2013). Similar studies using different sources of mineral water collected from depths lower than 0.5 km below the earth’s surface have demonstrated consistent results in improved muscle fatigue against an exercise challenge (Stasiule et al., 2014; Fan et al., 2016; Keen et al., 2016). The underlying mechanism to explain the significant outcomes on muscle recovery remains unclear.

Most of sports beverages contain fructose (ranged between 2–12%), which raises concern according to numerous studies reporting systemic and peripheral inflammations after prolonged fructose consumption (Wei et al., 2008). High fructose consumption increases TNF-α (Suehiro et al., 2016) and decreases GSH/GSSG ratio (Mariappan et al., 2007) in muscles. However, the fructose concentrations used in most of the previous studies were much higher than what people have actually consumed. It remains unclear whether consuming beverages with moderate fructose concentration would increase inflammation in exercised skeletal muscle.

Intensive muscle contraction during exercise can cause acute muscle inflammation, evidenced by leukocyte infiltration, and increased inflammatory cytokine levels (Bernecker et al., 2013). The inflammation process is essential to restore normal tissue function during recovery after a muscle damaging event (Tidball, 2017). During the early phase of inflammation, leukocytes infiltrate into challenged skeletal muscles (Yu et al., 2014). Phagocytic macrophages (CD68^+^, M1) emerge in the injured tissue to phagocytose unhealthy cells, concurrent with increases in TNF-α and reactive oxygen species (ROS) (Magalhães et al., 2012; Huang et al., 2017). Following phagocytosis, a protracted presence of regenerative macrophages (CD163^+^, M2) in recovering tissues is required to promote cell regeneration until resolution of inflammation (Villalta et al., 2008). The magnitude and duration of the entire inflammation process reflects the efficiency of post-exercise fatigue recovery. It has recently been shown that DOM supplementation attenuated the pro-inflammatory cytokine (TNF-α) response of adipose tissue in mice induced by a high-fat diet (Ha et al., 2014a). In this study, we aimed to answer the question whether prolonged DOM supplementation can inhibit inflammatory response of skeletal muscle after an acute bout of eccentric exercise in terrestrial animals regularly consuming 11% fructose beverage.

## Materials and Methods

### Animals

A total of 47 male Sprague Dawley (SD) rats (body mass 315 ± 6 g), 2 months of age, were obtained from LASCO Corporation (I-Lan, Taiwan). All rats were habituated to the housing environment in the Animal Center of University of Taipei (Taipei, Taiwan) for 7 days with no intervention. Two animals were housed per cage with standard laboratory chow available *ad libitum* (PMI Nutrition International, Brentwood, MO, United States) in an animal room with automated 12/12 h light/dark cycle, 22 ± 2°C, and 50% relative humidity. This study was approved by the Animal Care and Use Committee at University of Taipei (approval number 20120006), and conducted in accordance to Taiwan’s Animal Protection Act.

### DOM-Containing Fructose Drink

The DOM used in this study was supplied by Taiwan Yes Corporation (Hualien, Taiwan). Drinks were made from liquid concentrate of desalinated seawater collected from a depth of 662 m below the earth’s surface in the West Pacific Ocean, Hualien, Taiwan. DOM concentrate (D-MINNERALZ^®^) at a hardness of 170,000 mg/L was diluted with fructose (11%) to reach a hardness of 600 mg/L. The concentration of DOM used in the study was based on our previous study, which demonstrated an accelerated recovery from physical fatigue in humans (Hou et al., 2013). **Table 1** shows the mineral and trace element profile of the DOM-containing fructose drink at a hardness of 600 mg/L.

**Table 1:** Mineral and trace element profile of deep ocean mineral (DOM)-containing drink.

Mineral	DOM (mg/L)	Control (mg/L)	Trace element	DOM (μg/L)	Control (μg/L)
Ca	16	14	Li	44	24
Mg	165	0.8	Rb	64	50
K	39	0.3	B	4725	100
SO_4_	185	34	Si	<370	ND
Cl	518	ND	Al	<185	ND
Br	9	<0.5	Mn	<37	ND
Na	109	9.8	Fe	<74	ND
NO_3_	<37	ND	Ba	<37	ND
NH_4_	<0.02	ND	Mo	1.0	ND
NO_2_	<0.7	ND	As	<37	ND
PO_4_	0.75	0.69	Cd	<3.7	ND
F	0.54	<0.50	Co	<37	ND
P	0.02	ND	Ni	2.3	<2
H_2_S	<0.04	ND	Se	<37	ND
TC	0.56	ND	Sn	<37	ND
I	0.20	<0.2	Cs	<37	ND
La	<0.01	ND	Sb	<19	ND
Ce	<0.01	ND	Ga	<37	ND
Pr	<0.01	ND	Sr	59	38
Nd	<0.01	ND	Zn	<74	ND
Sm	<0.01	ND	Pb	<18.5	ND
Eu	<0.01	ND	V	2.0	0.52
Gd	<0.01	ND	Ag	<37	ND
Tb	<0.01	ND	Ti	<37	ND
Dy	<0.01	ND	Ge	<37	ND
Ho	<0.01	ND	Zr	<37	ND
Er	<0.01	ND	Nb	<37	ND
Tm	<0.01	ND	Ru	<37	ND
Yb	<0.01	ND	Rh	<37	ND
Lu	<0.01	ND	Pd	<37	ND
Y	<0.01	ND	Te	<37	ND
Sc	<0.01	ND	Dy	<37	ND
Th	<0.01	ND	Hf	<37	ND
In	<0.07	ND	Ta	<37	ND
P	<1	ND	W	7.6	ND
Bi	<0.04	ND	Re	<37	ND
			Os	<37	ND
			Ir	<37	ND
			Pt	<37	ND
			Au	<37	ND
			Tl	<37	ND
			U	<37	ND
			Cu	<18.5	ND
			Cr	<18.5	ND
			CO_3_	<22	ND

*ND, not detectable.*

### Experimental Design

Rats were randomized into the following 4 groups: Control (C, *N* = 12), Fructose (F, *N* = 12), Fructose+Exercise (FE, *N* = 12), Fructose+Exercise+DOM (FED, *N* = 11). In all fructose-treated groups, 11% fructose was freely accessible in a drinking bottle for 9 weeks. The concentration and duration of fructose supplementation were selected according to a previous study, which has shown an increased hepatic TNF-α mRNA after 9 weeks of treatment (Veličković et al., 2013). A longer period of DOM supplementation has been shown to decrease TNF-α response of adipose tissue in mice consuming a high-fat diet (Ha et al., 2014a). Food was provided *ad libitum* for all animals until 12 h before blood and muscle sample collection. Body mass of the rats amongst the 4 groups was not significantly different at the beginning of the study. Body mass and energy consumption (food and drink) were recorded every week at the same time of day throughout the study. Animals were acclimated to the exercise protocol on a rat treadmill for 10 min/day at a speed of 10 m/min (0°decline) for 1 week prior to the eccentric exercise challenge. Rats in the exercise groups (FE and FED) were challenged by 18-sessions of intermittent downhill running at a speed of 16 m/min (16°decline) for 5 min with 2 min rest interval between each bout of running. The overall duration of the exercise program was 90 min according to previously published methods (Yu et al., 2014). 24 h after exercise, rats were anesthetized at the same time with non-exercised rats for muscle tissue collection. Food and drink were temporarily removed from all rats during exercise.

### Tissue Collection

All rats were anesthetized using Zoletil (40 mg/kg BM) via intraperitoneal injection. A portion of soleus muscle was fixed in formalin, embedded in paraffin and then sliced to get 2-μm-thick sections for histological and immunohistochemical analyses. The rest of the soleus muscles were immediately frozen by liquid nitrogen and stored at −80°C until analyses.

### Reduced and Oxidized Glutathione

A glutathione fluorometric assay kit (Biovision, Milpitas, CA, United States) was used to detect reduced glutathione (GSH) and oxidized glutathione (GSSG) levels. Briefly, approximately 40 mg of muscle tissue was homogenized on ice with 100 μl of ice-cold glutathione assay buffer provided in the kit. Muscle homogenate was assayed according to manufacturer instructions, using an ELISA reader (Tecan GENios, A-5082, Tecan, Salzburg, Austria). Total protein content was determined using a BioRad Protein Assay reagent (BioRad, Hercules, CA, United States). Concentration of GSH and GSSG were standardized by protein content of each sample and expressed as ng per mg of protein.

### Antioxidant Enzyme Activities

Muscle samples (40–50 mg) were homogenized at a 1:10 ratio (W/V) in ice cold buffer containing 50 mM Tris–HCl, pH 7.5 with 1 mM EDTA, using a polytron homogenizer, and centrifuged at 10,000 g for 10 min at 4°C. The resulting supernatant was collected for analyses. Spectrophotometric assay kits were used to measure glutathione peroxidase (GPx) and catalase (CAT) (Cayman Chemical Company, Ann Arbor, MI, United States) activity levels. Both GPx and CAT activities were measured in accordance with the protocol supplied by the manufacturer. Total protein content was determined using a BioRad Protein Assay reagent (BioRad, Hercules, CA, United States). The activity levels were standardized by protein content of each sample and expressed as nmol per min per mg protein.

### Histology and Immunohistochemistry (IHC)

Histology and immunohistochemistry analyses were conducted by a certified pathologist at China Medical University Hospital (Taichung, Taiwan). Muscle cross-sections were stained with hematoxylin and eosin (H and E) to detect the amount of leukocyte infiltration and centrally nucleated fibers (marker for newly regenerated muscle fibers). The values were expressed as a percentage of the total number of fibers in the muscle cross-section by counting complete cross-sections of soleus muscles from all animals to provide an index of injured fibers and muscle regeneration. Immunohistochemistry was performed to visualize macrophages in invaded tissues. Briefly, soleus muscle was fixed in 4% buffered formalin and embedded in paraffin. Tissue sections were cut in 2 μm slices and transferred onto coated slides (Super Frost Plus, Braunschweig, Germany). Antigen retrieval occurred in boiled water for 15 min in 0.1 M sodium citrate (pH 7.2). These pretreated slides were blocked for 15 min at room temperature with 5% BSA and then incubated at 4°C overnight with primary antibodies: antibodies against rat CD68 (dilution 1:100) (abcam, Cambridge, United Kingdom) and rabbit CD163 (dilution 1:100) (AbD Serotec, Kidlington, United Kingdom). Specific antibody was purchased to perform IHC staining by using horseradish peroxidase-conjugated avidin biotin complex (ABC) from the Vectastain Elite ABC Kit (Vector Laboratories, Burlingame, CA, United States) and DAB chromogen (Vector Laboratories). The stained sections were captured using a light microscope (Olympus BX51, Olympus Corporation, TKY, Japan) with a 40X objective (magnification: 400X). Five areas of random field (80–100 mm^2^), encompassing > 500 fibers, were scanned for each section. The inflammation grade, CD68 and CD163 positive cells were evaluated by a semi-quantitative scoring method as previously described (Zlobec et al., 2006). The expression levels were scanned overall staining intensity and scored by an experienced pathologist who was blinded to treatment. Semi-quantitative scores were subsequently categorized by a scoring system at 4 levels, score 0: no cluster of positive cells; score 1: 1–3 clusters of positive cells; score 2: 4–6 clusters of positive cells; and score 3: >7 clusters of positive cells.

### Quantitative Polymerase Chain Reaction (PCR)

Total RNA was extracted from the frozen muscles in ice-cold TriReagent (Sigma-Aldrich, St. Louis, MO, United States), according to the manufacturers guidelines. Extracted RNA was quantified at 260 nm, and the 260/280 ratio (1.6∼2) was used to verify the purity of RNA. Reverse transcription was then performed with iScript cDNA Synthesis Kit (BioRad, Hercules, CA, United States) to generate cDNA according to the manufacturer’s recommendations. Real-time PCR was performed in iQ Supermix (BioRad, Hercules, CA, United States), primers and TaqMan probes (Sigma-Aldrich, Singapore) in MyiQ Single Color Real-Time PCR Detection System. The following primer and probe sequences were used for amplification of target genes: COX-2 (NM_017232) forward primer: CAGTCTCTCATCTGCAATA; reverse primer: AGGGTTAATGTCATCTAGTC; probe:TCCCTTTGCCTCTTTCAATGTGC. TNF-α (NM_012675) forward primer: GAGTCATTGCTCTGTGAG; reverse primer: CTCTGAGGAGTAGACGATA; probe: CTGGCGTGTTCATCCGTTCTCT. IL-10 (L02926) forward primer: GATCCAGAGATCTTAGCTA; reverse primer: CTGAGGTATCAGAGGTAA; probe: AACCTCGTTTGTACCTCTCTCCAA, where 18S gene was included as internal housekeeping control. Samples from each group were run in duplicate on the same plate. The relative quantification of gene expression was calculated using the efficiency prior from series diluted standard curve. Comparative cycle threshold (Ct) calculations for genes of interest were expressed relative to the 18S generated from the same cDNA sample. Data were normalized to the housekeeping gene (18S rRNA), and were expressed as the fold of the control value.

### Western Blotting Analysis

Muscle samples weighing 80–100 mg were homogenized in a 1:8 ratio of ice-cold homogenizing buffer containing 20 mM Hepes, 1 mM EDTA, and 250 mM sucrose, using a Polytron homogenizer (Fisher Scientific, Taipei, Taiwan), and centrifuged at 10,000 *g* for 10 min at 4°C. The resulting supernatant was collected, and total protein content was determined by a BioRad Protein Assay reagent (BioRad, Hercules, CA, United States). Equal amounts of protein, 50 μg, were loaded on 8–12% SDS-polyacrylamide gels and transferred to nitrocellulose (NC) membranes (BioRad, Hercules, CA, United States). The blots were blocked 1 h in 1X TTBS with 7% dry skim milk at room temperature. After the washing step, membranes were probed overnight at 4°C with primary antibody against nitrotyrosine (Millipore, Bedford, MA, United States, dilution 1:1000); antibodies against iNOS and eNOS (BD Transduction Laboratories, Bedford, MA, United States, dilution 1:1000); or antibody against GAPDH (Sigma, St Louis, MO, United States, dilution 1:5000), served as an internal control. Antibody-bound protein was detected using a peroxidase-conjugated anti-mouse secondary antibody (Sigma, St Louis, MO, United States, dilution 1:20,000) or anti-rabbit IgG (Cell Signaling Technology, Beverly, MA, United States, dilution 1: 2000). Protein signals were visualized using the enhanced chemiluminescent system (PerkinElmer Life and Analytical Sciences, Shelton, CT, United States). Band intensities were quantified using ImageJ software^1^.

### Statistical Analysis

A one-way analysis of variance (ANOVA) was conducted to compare the differences of mean among groups on all variables. Duncan *post hoc* test was used to distinguish the difference of mean between pair of groups. All values are expressed as mean±standard error (SE). A level of *P* < 0.05 was set for statistical significance for all tests.

## Results

**FIGURE 1:**
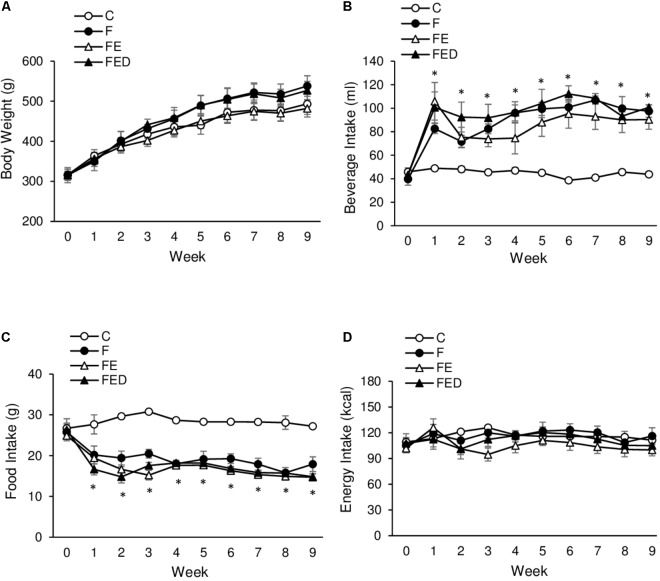
Body mass and energy intake. Body mass among the 4 groups was not statistically different **(A)**. Fructose-treated groups (11%) show greater beverage intake **(B)** and reciprocally lower food intake **(C)**, compared with the C group. No significant difference in total energy consumption was observed among the 4 groups **(D)**. Abbreviation: C, Control; F, Fructose; FE, Fructose+Exercise; FED, Fructose+Exercise+Deep ocean minerals (DOM). ^∗^Fructose-treated groups (F, FE, and FED) compared against the C group, *P* < 0.05.

Body mass was not significantly different among the 4 groups throughout the study (**Figure 1A**). Soleus muscle mass was also similar among the 4 groups (C: 0.25 ± 0.01 g; F: 0.30 ± 0.04 g; FE: 0.24 ± 0.01 g; FED: 0.24 ± 0.01 g). Drink intake in the fructose-treated groups (F, FE, and FED) was significantly higher than that in the C group (**Figure 1B**, *P* < 0.05), whereas food intake showed an opposing outcome (**Figure 1C**, *P* < 0.05). No significant difference in total energy intake was observed among the 4 groups (**Figure 1D**).

**FIGURE 2:**
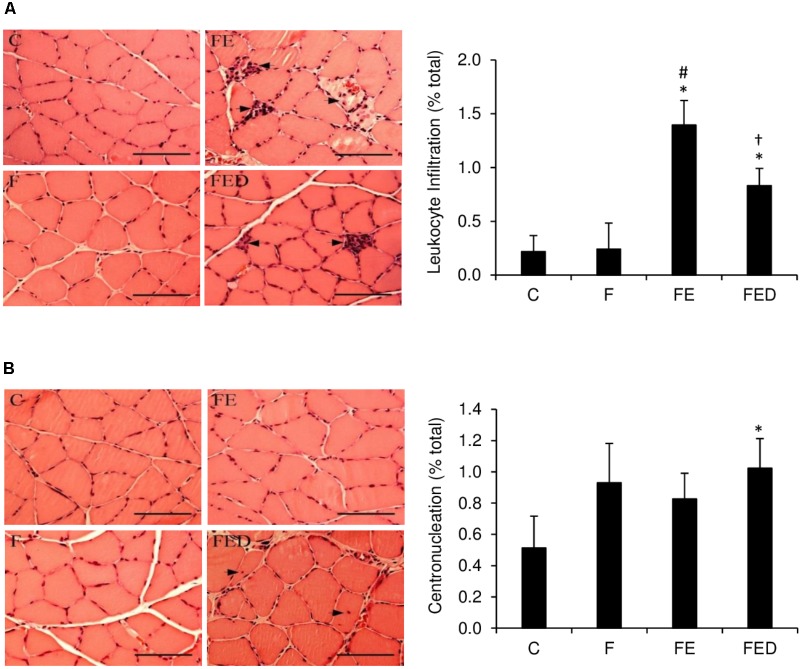
Leukocyte infiltration and centronucleation 24 h after downhill running. Representative hematoxylin and eosin (HandE) staining images of soleus muscle were shown on the left side of the bar chart (infiltrated leukocyte and centrally nucleated fibers indicated by arrowhead). The values are expressed as percentage of total fibers in the muscle cross-section. Leukocyte infiltration to soleus muscle in the FED group was lower than that in the FE group **(A)**. Centrally nucleated fibers in the FED group were higher than that in the C group **(B)**. Original magnification: 400 x. Scale bar: 100 μm. Abbreviation: C, Control; F, Fructose; FE, Fructose+Exercise; FED, Fructose+Exercise+Deep ocean minerals (DOM). ^∗^Compared against the C group, *P* < 0.05; ^#^Compared against the F group, *P* < 0.05; ^†^Compared against the FE group, *P* < 0.05.

**FIGURE 3:**
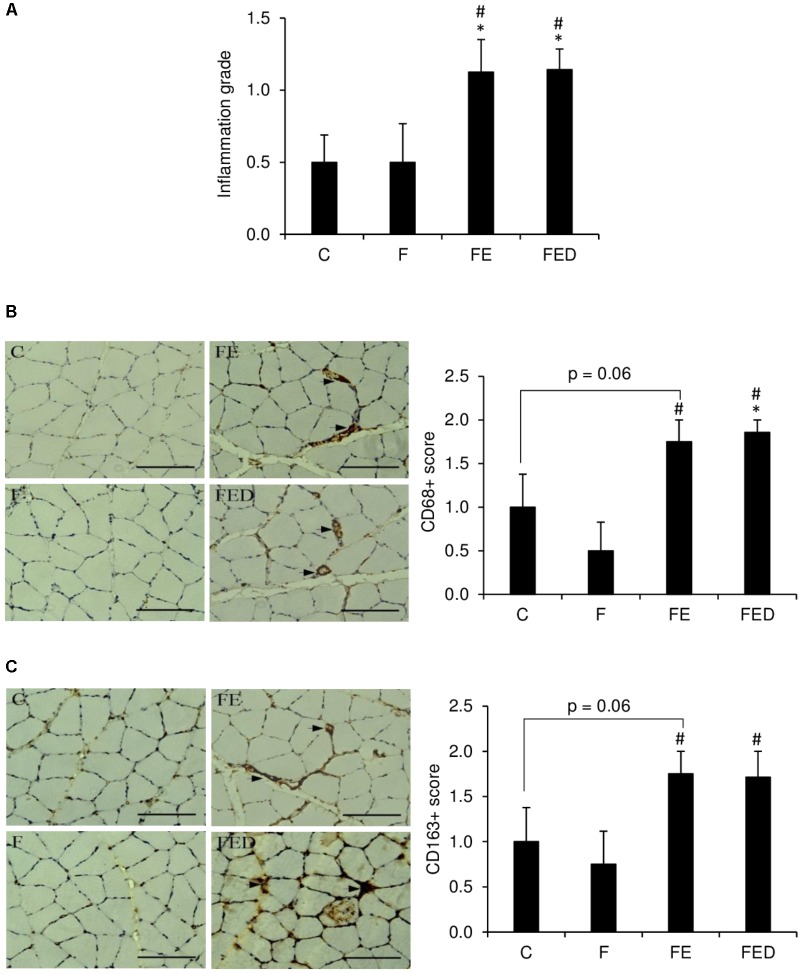
Macrophage infiltration 24 h after downhill running. Immunohistochemistry staining images are illustrated on the left side of bar chart (invaded macrophages indicated by arrowhead). Exercised groups (FE and FED) showed greater inflammation grade **(A)**, M1 (CD68^+^) **(B),** and M2 (CD163^+^) **(C)** macrophages in soleus muscle than non-exercised groups (C and F). No differences on these markers were detected between the FE and FED groups. The inflammation grade, CD68, or CD163 positive cells were evaluated by a semi-quantitative scoring method categorized into 4 categories, score 0: no cluster of positive cells; score 1: 1–3 clusters of positive cells; score 2: 4–6 clusters of positive cells; score 3: >7 clusters of positive cells. Abbreviation: C, Control; F, Fructose; FE, Fructose+Exercise; FED, Fructose+Exercise+Deep ocean minerals (DOM). ^∗^Compared against the C group, *P* < 0.05; ^#^Compared against the F group, *P* < 0.05.

To examine whether DOM can influence exercise-induced muscle inflammation in rats regularly receiving fructose drink, leukocyte infiltrations, centronucleation (**Figure 2**) and macrophage invasions (**Figure 3**) in the soleus muscle were measured 24 h after exercise. Leukocyte infiltration (**Figure 2A**), inflammation grade (**Figure 3A**), CD68^+^ M1 macrophage (**Figure 3B**), and CD163^+^ M2 macrophage (**Figure 3C**) in muscle of the FE group were 600%, 120%, 260%, and 125% greater than those of the F group (*P* < 0.05). Leukocyte number in muscle of the FED group was 43% lower than that of the FE group (FE: 1.4 ± 0.4% total; FED: 0.8 ± 0.3% total, *P* < 0.05). No significant differences in inflammation grade, CD68^+^ M1 macrophage, and CD163^+^ M2 macrophage between the FE and FED groups were detected. Centrally nucleated fibers in muscle of the FED groups were 50% greater than that of the C group and were similar to the F and FE groups (C: 0.5 ± 0.2% total; F: 0.9 ± 0.3% total; FE: 0.8 ± 0.2% total; FED: 1.0 ± 0.2% total, *P* < 0.05, **Figure 2B**). No significant DOM effect was observed.

**FIGURE 4:**
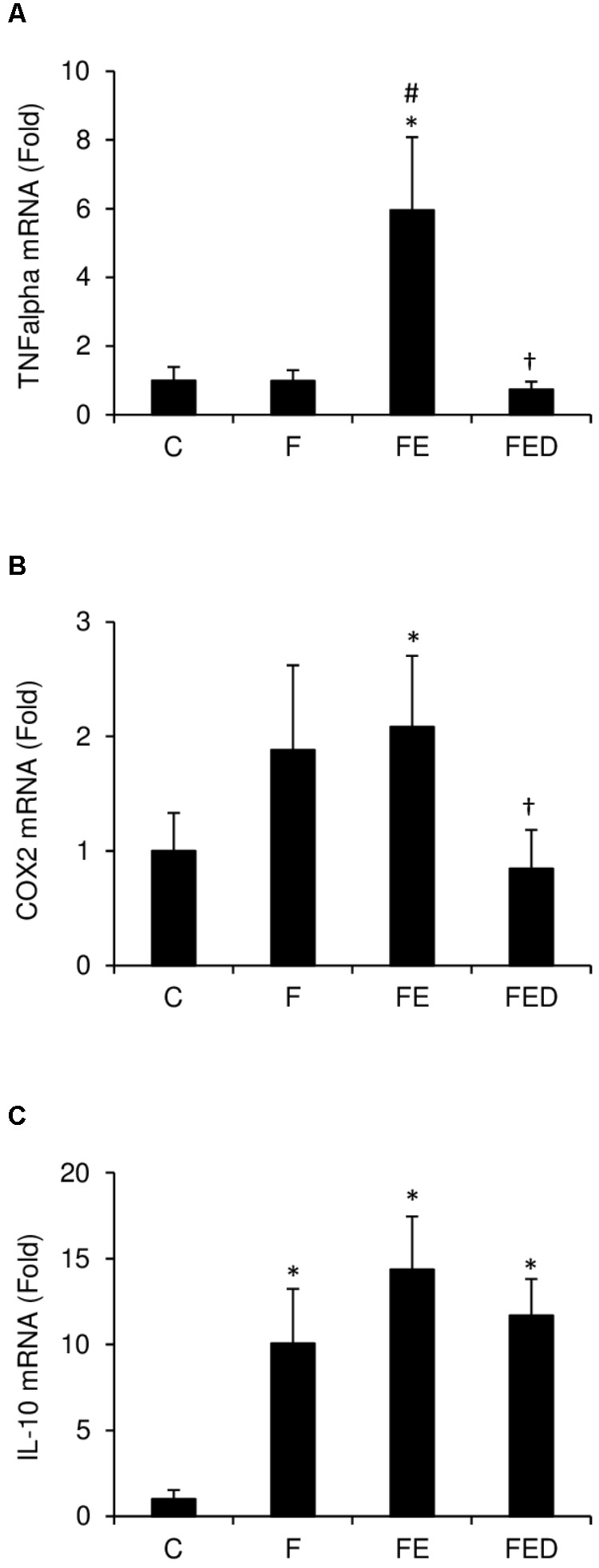
Inflammatory mediator expression 24 h after downhill running. Data were normalized to 18S rRNA, and were expressed as fold of the C group. TNF-α mRNA **(A)** and COX-2 mRNA **(B)** of soleus muscle in the FE group were greater than those in the C group. Both markers were similar for the C and FED groups. Fructose-treated groups (F, FE, and FED) showed greater IL-10 mRNA level **(C)** than the C group. Abbreviation: C, Control; F, Fructose; FE, Fructose+Exercise; FED, Fructose+Exercise+Deep ocean minerals (DOM); TNF-α, tumor necrosis factor-α; COX-2, cyclooxygenase-2; IL-10, interleukin-10. ^∗^Compared against the C group, *P* < 0.05; ^#^Compared against the F group, *P* < 0.05; ^†^Compared against the FE group, *P <* 0.05.

**FIGURE 5:**
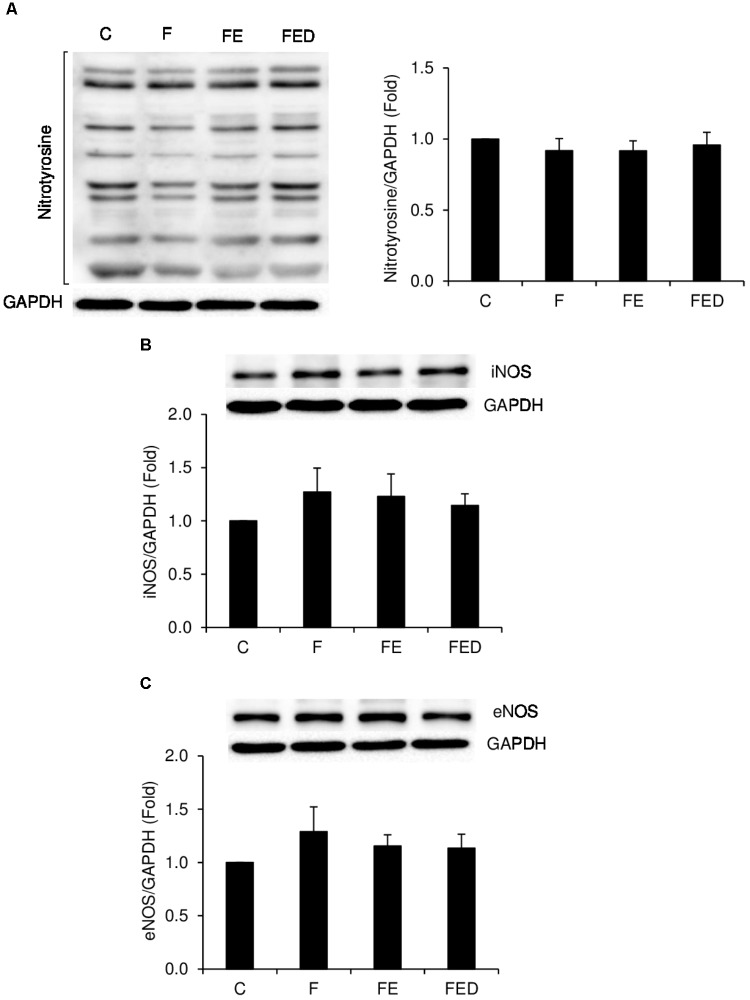
Nitrotyrosine, iNOS, and eNOS protein levels 24 h after downhill running. Representative images of western blots are illustrated on the left side or the top of the bar chart. Nitrotyrosine **(A)**, iNOS **(B)**, and eNOS **(C)**, normalized to glyceraldehyde-3-phosphate dehydrogenase (GAPDH), were similar among the 4 groups. Abbreviation: C, Control; F, Fructose; FE; Fructose+Exercise; FED, Fructose+Exercise+Deep ocean minerals (DOM); iNOS, inducible nitric oxide synthase; eNOS, endothelial nitric oxide synthase.

TNF-α mRNA (**Figure 4A**) in muscle of the FE rats were ∼five fold higher than those of the F groups (*P* < 0.05). TNF-α mRNA and COX-2 mRNA (**Figure 4B**) in muscle of the FED group were 88 and 62%lower, respectively, than those of the FE group (*P* < 0.05). IL-10 mRNA levels (**Figure 4C**) of all fructose-supplemented groups were 9–13 times greater than that of the C group (*P* < 0.05). In the study, nitrotyrosine (**Figure 5A**), iNOS protein (**Figure 5B**), and eNOS protein (**Figure 5C**), measured 24 h after exercise, were similar among the 4 groups.

**FIGURE 6:**
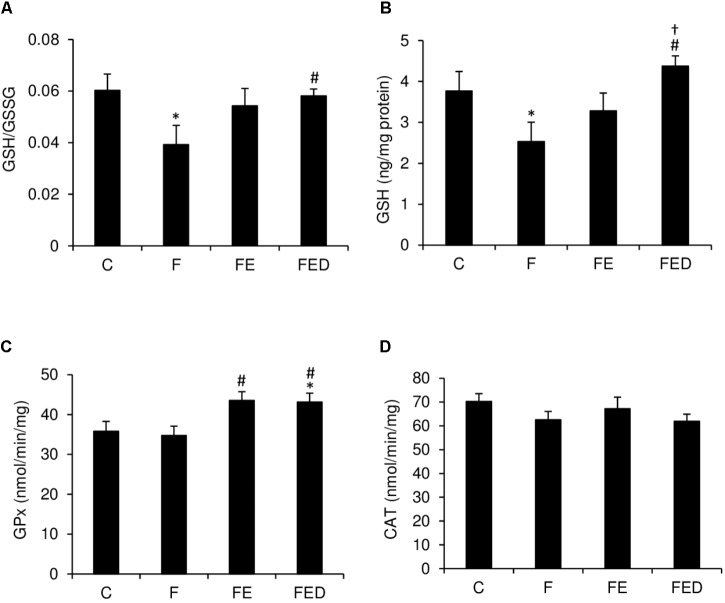
Redox status and antioxidant enzyme activity 24 h after downhill running. GSH/GSSG ratio **(A)** and GSH **(B)** of soleus muscle in the F group were significantly lower than those in the C group. Both markers were similar for the C and FED groups. Exercised groups (FE and FED) show moderately greater GPx activity **(C)** than the C group. CAT activity **(D)** was similar among the 4 groups. Abbreviation: C, Control; F, Fructose; FE, Fructose+Exercise; FED, Fructose+Exercise+DOM; GSH, reduced glutathione; GSH/GSSG ratio, reduced to oxidized glutathione ratio; GPx, glutathione peroxidase; CAT, catalase. ^∗^Compared against the C group, *P* < 0.05; ^#^Compared against the F group, *P* < 0.05; ^†^Compared against the FE group, *P <* 0.05.

Redox state and antioxidant enzyme activity of muscle are shown in **Figure 6**. After 9 weeks of fructose drinking, GSH/GSSG ratio (**Figure 6A**), and GSH (**Figure 6B**) and in muscle of the F group were 40 and 33% lower, respectively, than those of the C group (*P* < 0.05). However, GSH/GSSG ratio and GSH in the FED group were 33 and 76% greater, respectively, than those in the F group (*P* < 0.05). Moreover, GSH level in the FED group was 25% greater than that in the FE group (*P* < 0.05). GPx activity (**Figure 6C**) in muscle of the exercised groups (FE and FED) was significantly greater than that of the F group (*P* < 0.05). No DOM treatment effect was detected for both GPx and CAT activities (**Figure 6D**).

## Discussion

Fructose is a widely used ingredient in sports beverages. Previous studies suggest that fructose causes systemic inflammation (Jameel et al., 2014). However, the fructose concentration used in commercial beverages generally falls within the range of 2–12%, which is substantially lower than most of the previous studies reporting inflammation. In the present study, the acute inflammatory response in skeletal muscle after downhill running was measured in rats regularly consuming a fructose-containing drink (11%) with and without addition of DOM. We found significant elevations in IL-10 mRNA (9–12 times of the C group) in muscle from rats consuming the fructose drink. Furthermore, eccentric exercise significantly increased leukocyte infiltration and TNF-α mRNA in the muscle tissue. DOM has been reported to suppress increases in plasma TNF-α protein of diabetic mice (Ha et al., 2014b) and adipose tissue TNF-α mRNA of obese mice (Ha et al., 2014a). The results of the study suggest that addition of DOM into the drink significantly attenuated the acute responses in leukocyte infiltration and TNF-α mRNA of exercised skeletal muscle for rats regularly consuming a fructose drink. TNF-α mRNA is generally increased during the early phase of muscle inflammation after a physical challenge (Tidball and Villalta, 2010). Thus, the suppressed response in TNF-α mRNA and leukocyte infiltration of exercised skeletal muscle with DOM supplementation implicates an accelerated resolution of muscle inflammation after, and potentially during, eccentric exercise.

It is intriguing that there was a significant difference in leukocyte content between FE and FED, but no difference in inflammation grade, CD68^+^ macrophage, and CD163^+^ macrophage between the two groups were detected. We must highlight that only a very small fraction of leukocytes develop into macrophages. In particular, macrophages are differentiated from monocytes (blood, and spleen), which only accounts for 2–10% of circulating leukocytes (Italiani and Boraschi, 2014). The observed results suggest that other sub-types of leukocytes (e.g., neutrophils) also contributed to the repair process after the exercise challenge (Tidball, 2017).

Challenged with the same eccentric exercise protocol for rats without prolonged fructose supplementation, our previous study has shown increased inflammation, necrotic fibers, and nitrotyrosine with no centronucleation (Huang et al., 2017). Most of the results in the study are consistent with our previous observations. However, in the present study we found no differences in nitrotyrosine levels between the exercise and non-exercise groups. The discrepancy for the divergent results on nitrotyrosine levels may have been associated with a dramatic elevation in IL-10 mRNA by prolonged fructose supplementation in the present study. IL-10 is known to inhibit nitric oxide production of phagocytic macrophages (Baseler et al., 2016) and to increase muscle regeneration (Tidball, 2017). In this study, the increases in muscle IL-10 mRNA in the fructose-fed rats after exercise fit well with the observation with no increase in nitrotyrosine levels. Given that the soleus muscle mass was similar for the control and fructose-fed groups, increases in IL-10 mRNA suggest an increased nucleus turnover in muscle tissue. Furthermore, we did not observe significant changes in iNOS and eNOS protein levels in the exercised muscle. Eccentric exercise is known to induce iNOS and eNOS protein levels within the muscle in 2 h (Lima Cabello et al., 2010). Therefore, no responses in nitrotyrosine, iNOS and eNOS protein levels of exercised muscle indicate that the nitric oxide production mechanism during exercise-induced muscle inflammation is transient, and may have already returned to non-exercised levels from its peak 24 h after eccentric exercise.

Glutathione plays an essential role in maintaining intracellular redox state (Asensi et al., 1999). Decreases in GSH level and GSH/GSSG ratio in liver and brain have been reported after prolonged high-fructose supplementation (Song et al., 2012; Jarukamjorn et al., 2016; Sil and Chakraborti, 2016). A similar decrease in GSH/GSSG ratio of skeletal muscle after long-term fructose drinking in this study indicates that the observed effect on redox state is systemic. The results of the present study suggest that exercise combined with DOM can attenuate the GSH-lowering effect of fructose. The pattern of the changes in GSH among the 4 treatment groups appears to be unrelated with antioxidant enzyme GPx and CAT. Both enzymes function to eliminate ROS by decomposing the toxic hydrogen peroxide (H_2_O_2_) to water and oxygen. GPx activity is known to increase after an acute bout of exercise both in human (Berzosa et al., 2011) and animal (Radak et al., 1995; Hsu et al., 2017). In this study we found a small increased GPx after exercise, but no change in CAT was detected. This is probably due to the time of muscle tissue collection.

A noteworthy feature of DOM is enriched minerals (such as magnesium) and trace elements (such as lithium, rubidium, and boron) compared with inland and ocean surface. Components in DOM may have been associated with strengthened antioxidant property against ROS. For example, magnesium supplementation has been reported to lower free radicals concentration induced by electric shocks (Zhang et al., 2003) and ischemic reperfusion in animals (Garcia et al., 1998). Boron can reduce magnesium loss (Nielsen et al., 1987), minimize TNF-α level (Naghii et al., 2011), and increase ROS scavenging capacity in animals (Bhasker et al., 2016). Lithium administration is known to increase GSH, decrease TNF-α, and minimize ROS in animals (Albayrak et al., 2013). Given that ROS is mostly generated during inflammation, the lowered inflammatory response against the exercise challenge observed in the present study is likely associated with the action of minerals and trace elements in DOM.

The major limitation of the study is that DOM used in this study comes from the ocean water at the depth of 662 m below the earth’s surface. The mineral and trace element profile of DOM are subject to change in accordance with the depth of the ocean below the earth’s surface (Kastner, 1999). Therefore, whether the DOM collected from different depths in the ocean can produce similar effects on muscle inflammation remains unresolved.

## Conclusion

The results of the present study demonstrate a significant influence of prolonged fructose drinking on the inflammatory balance of skeletal muscle. Furthermore, this study provides encouraging evidence suggesting that the components in DOM can minimize inflammatory cytokine responses of skeletal muscle after eccentric exercise.

## Author Contributions

SS and C-HK had full access to all of the data in the study and took responsibility for the integrity of the data and the accuracy of the data analysis. SS, RC, and C-HK carried out the study concept and design. SS and C-HK drafted the manuscript. SS, JW, C-YH, C-LK, MH, RC, and C-HK carried out the critical revision of the manuscript for important intellectual content. All authors have read and approved the final version of the manuscript and agreed with the order of presentation of the authors.

## Conflict of Interest Statement

The authors declare that the research was conducted in the absence of any commercial or financial relationships that could be construed as a potential conflict of interest.

## Footnotes

1 http://rsb.info.nih.gov/ij/
